# Motion cues modulate responses to emotion in movies

**DOI:** 10.1038/s41598-018-29111-4

**Published:** 2018-07-18

**Authors:** Eran Dayan, Avi Barliya, Beatrice de Gelder, Talma Hendler, Rafael Malach, Tamar Flash

**Affiliations:** 1 Department of Radiology and Biomedical Research Imaging Center, University of North Carolina at Chapel Hill, Chapel Hill, NC USA; 2 Department of Computer Science and Applied Mathematics, Weizmann Institute of Science, Rehovot, Israel; 3 Brain and Emotion Laboratory, Maastricht University, Maastricht, The Netherlands; 4 Tel Aviv Center For Brain Functions, Tel Aviv Sourasky Medical Center, Tel Aviv, Israel; 5 Department of Neurobiology, Weizmann Institute of Science, Rehovot, Israel

## Abstract

Film theorists and practitioners suggest that motion can be manipulated in movie scenes to elicit emotional responses in viewers. However, our understanding of the role of motion in emotion perception remains limited. On the one hand, movies continuously depict *local motion*- movements of objects and humans, which are crucial for generating emotional responses. Movie scenes also frequently portray *global motion*, mainly induced by large camera movements, global motion being yet another source of information used by the brain during natural vision. Here we used functional MRI to elucidate the contributions of local and global motion to emotion perception during movie viewing. Subjects observed long (1 min) movie segments depicting emotional or neutral content. Brain activity in areas that showed preferential responses to emotional content was strongly linked over time with frame-wide variations in global motion, and to a lesser extent with local motion information. Similarly, stronger responses to emotional content were recorded within regions of interest whose activity was attuned to global and local motion over time. Since global motion fields are experienced during self-motion, we suggest that camera movements may induce illusory self-motion cues in viewers that interact with the movie’s narrative and with other emotional cues in generating affective responses.

## Introduction

A key determinant for the popularity and success of movies lies in their capability to stimulate and entertain viewers by effectively modulating their emotional states. While content and narrative are the most obvious modulators of viewers’ emotional responses they are rarely manipulated in isolation during movie scenes. Rather, movie directors use a variety of cinematographic techniques that supplement and enhance the movie’s narrative and content in eliciting emotional responses in viewers^1^. As such, film stimuli provide a compelling way to study how a range of factors mediate emotion perception. Among the most powerful and basic of the cinematographic techniques used to elicit affective responses is the manipulation of movement^1,2^. Two sources of motion are of particular interest, namely global and local motion^3^. On the one hand, movies continuously depict local motion- the movement of various types of objects, including humans- which may convey essential information that combines facial expression and bodily movements in generating an emotional percept^4,5^. Movie scenes also continually portray global motion^3^, another powerful source of motion information, mainly induced by large camera movements, where the entire scene moves. The global flow fields induced by camera movement resemble those experienced by humans during self-motion^3^, and may thus tap into another source of information used by the brain during natural vision^6–8^ and social interaction settings^9^. While film theorists and cinematographers have noted the central role that manipulation of motion plays in eliciting emotional responses in movie viewers^1,2^ very little is known about the mediating role of motion in emotion perception. In particular, a mechanistic understanding of the contribution of local and global motion information to emotion perception in the context of the rich and realistic visual settings of movie viewing^10^ is missing. To address this question we asked participants to observe a series of uninterrupted 1-min long clips portraying emotional or emotionally neutral content while recording subjects’ brain activity using functional MRI. Dynamic frame-by-frame fluctuations in global and local motion were then quantified from the movie stimuli, testing the involvement of these two motion signals in emotion–induced brain activity.

## Results

**Figure 1:**
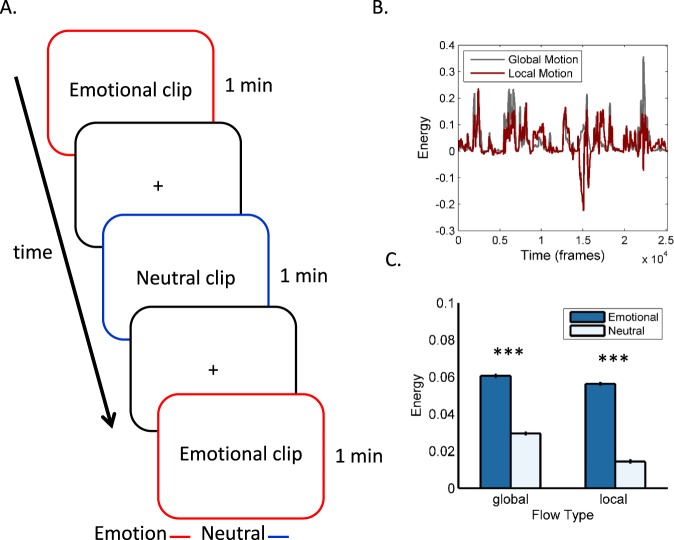
Experimental design and stimuli. (**A**) Blood oxygenation level dependent (BOLD) responses were recorded while subjects observed 1-min long clips, interleaved by short periods of fixation, depicting either emotional or emotionally neutral content. The clips were extracted from popular movies (see Materials and Methods). Motion information contained in the clips was quantified by computing frame-wide changes in optical flow and partitioning these into changes in global and local motion fields. The global and local motion signals were tightly correlated across time (**B**) and were stronger during emotional than during emotionally neutral movie frames (**C**).

Subjects were asked to observe a series of 1-min long clips portraying emotional or emotionally neutral content, interleaved by short periods of fixation (Fig. 1A). The clips were extracted, with no sound, from popular commercial movies (see Materials and Methods) and typically depicted complex interactions between 2 actors or more. Emotional clips contained emotions such as anger, joy or a combination of anger and another emotion (e.g., an interaction between an angry protagonist and another fearful actor or actress). Emotionally neutral clips were extracted from the same movies and displayed human movements and actions, but no emotional content. The clips were chosen from a larger pool of clips based on ratings from six observers (mean age = 29.5, 4 females, none of which participated in the imaging experiment). To assess the contribution of global and local motion, we quantitatively tracked scene-wide changes in optical flow and partitioned these changes into components related to global and local motion^3^. Both global and local motion continuously fluctuated across all 14 clips. The two signals were strongly correlated across time on a frame-by-frame basis (r = 0.403, p < 0.0001; Fig. 1B), both showing significantly higher amplitudes during emotional, relative to neutral clips (t = 42.26, p < 0.0001 and t = 58.89, p < 0.0001 for global and local motion frames respectively; Fig. 1C). This suggests that global and local motion did not occur in isolation, but rather that the signals co-varied across large portions of the movie clips.

### Links between local and global motion and emotion-related brain activity

**Figure 2:**
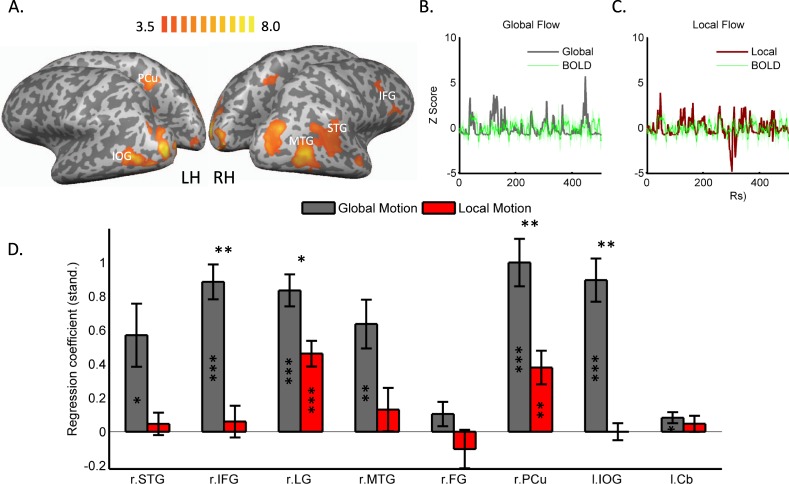
Brain activity associated with observation of emotion in movies. (**A**) Regions showing significantly stronger BOLD responses to emotional relative to neutral clips, FDR corrected for multiple comparisons. To assess the contribution of motion to the perception of emotion in movies the global (**B**) and local (**C**) motion time series were regressed with individual-subject BOLD signals extracted from each of the regions displayed in A, shown here for one representative region (color shades denote standard errors of the mean). (**D**) Brain activation in emotion responsive brain regions was strongly tuned to global and to a lesser degree to local motion. Asterisk signs within bars denote the results of one-sample t tests, indicating whether the regression coefficients were significantly different than ‘0’. Asterisk signs above bars denote the results of paired t tests, comparing links (regressions coefficients) between BOLD signals and global and local flow. Cb, cerebellum; FG, fusiform gyrus; IFG, inferior frontal gyrus; IOG, inferior occipital gyrus; Lg, lingual gyrus; MTG, medial temporal gyrus; Pcu, precuneus; STG, superior temporal gyrus; * indicates statistical significance at P < 0.05; **p < 0.01; ***P < 0.001. Significance estimates were FDR corrected for multiple comparisons.

Analysis of imaging data revealed significantly stronger blood oxygenation level dependent (BOLD) responses to emotional relative to neutral clips (Fig. 2A; Table 1) in right inferior frontal gyrus (IFG), bilateral occipito-temporal cortex, right superior temporal gyrus (STG), right precuneus (PCu), right fusiform gyrus, left inferior occipital gyrus (IOG) and left cerebellum. To probe for the contribution of motion to emotion-induced activation within these regions, we extracted BOLD signals from each of the regions and each of the subjects and regressed them with the global (Fig. 2B) and local (Fig. 2C) flow signals, which were down-sampled and convolved with a hemodynamic response function. Global motion showed a significant link (significant regression coefficients, corrected for multiple comparisons using FDR correction) with activations derived from most (6/8) of the emotion-responsive regions (Fig. 2D), including right STG, right IFG, right lingual gyrus (LG), right medial temporal gyrus (MTG), right PCu and left IOG. Local motion, on the other hand, showed overall significantly weaker links with BOLD activations derived from the same regions, with two regions (right LG and right PCu) showing significant links with local flow.

**Table 1:** Areas showing significantly stronger BOLD responses during emotional relative to neutral movie clips.

Region	Side	BA	X	Y	Z	t	p
Superior Temporal Gyrus/Sulcus	R	39	45	−49	25	5.96	0.000094
Inferior Frontal Gyrus	R	44	51	14	19	6.18	0.00007
Lingual Gyrus	R	17	21	−91	4	13.58	<0.00001
Middle Temporal Gyrus	R	21	51	−22	−8	6.16	0.000071
Fusiform Gyrus	R	19	21	−61	−8	6.39	0.000051
Precuneus	R	7	6	−46	46	6.13	0.000074
Inferior Occipital Gyrus	L	19	−42	−76	−2	11.46	<0.00001
Posterior cerebellum	L		−33	−37	−38	8.64	0.000003

Significance levels were corrected for multiple comparisons both at the voxel level (FDR < 0.05) and at the cluster level (at p < 0.05). BA, Brodmann area.

### Emotion-related activity in regions responsive to global and local motion

**Figure 3:**
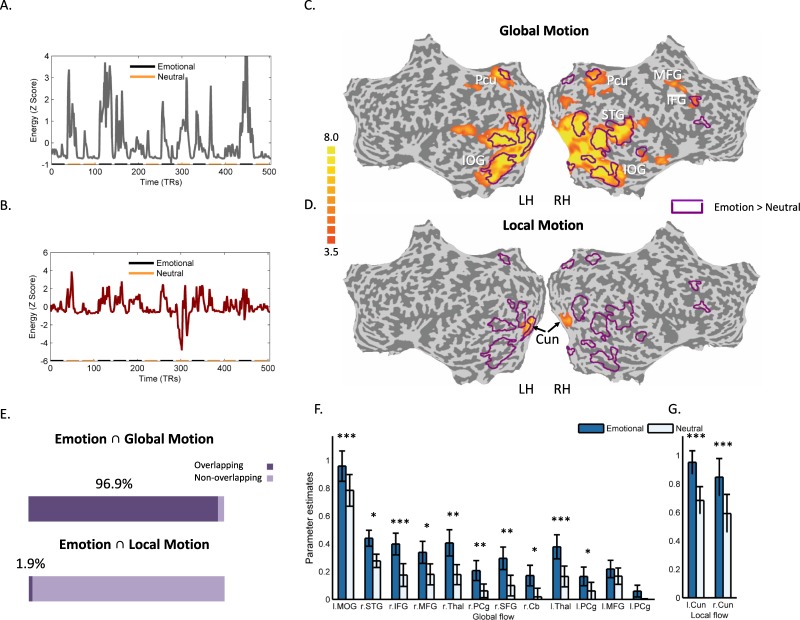
Analysis of brain activity induced by global and local flow. (**A**,**B**) The global (**A**) and local (**B**) flow signals served as a predictors in a GLM analysis. The signals are displayed along with the corresponding segments where emotional and neutral clips were shown. (**C**,**D**) Regions whose activity was correlated with the global (**C**) and with the local (**D**) flow predictors are shown in orange alongside the contour of regions that showed significantly stronger BOLD responses to emotional relative to neutral clips (as displayed in Fig. 2). The map is FDR corrected (<0.05) for multiple comparisons (**E**) Estimates for the overlap between emotion-related brain activations and those induced by global and local flow (**F**,**G**) Region of Interest analysis (ROI) comparing brain activity elicited by emotional and neutral content within all the regions responsive to global (**F**) and local flow (**G**). *Indicates statistical significance at P < 0.05; **P < 0.01; ***P < 0.001. Cu, Cuneus; MFG, middle frontal gyrus; MOG, Middle occipital gyrus; PCg, Posterior cingulate gyrus; SFG, Superior frontal gyrus; Thal, Thalamus. All other abbreviations are as in previous figures.

To provide additional evidence for the contribution of motion signals to the neural responses to emotion in movies we used the global (Fig. 3A) and local (Fig. 3B) motion time series as inputs in a general linear model (GLM) analysis, aiming to first identify regions that were responsive to these motion signals. Activation in a widely distributed set of brain regions was significantly correlated with global motion (Fig. 3C, Table 2), including areas in frontal, cingulate and occipito-temporal cortices. Regions responsive to local motion (Fig. 3D, Table 2), on the other hand, were more spatially restricted and were centered bilaterally in the cuneus. Overlaying the statistical map of emotion-responsive brain regions, identified in our previous analysis, on top of the map of areas responsive to global flow (purple contours in Fig. 3C) revealed a highly extensive degree of overlap among the two maps (Fig. 3E), further establishing the centrality of global motion signals in emotion perception. In contrast, the map of regions which were responsive to local flow, showed little overlap with emotion-responsive regions (Fig. 3D,E). We next performed region of interest (ROI) analysis within each of the regions that showed stronger responses to global (Fig. 3F) and local motion (Fig. 3G), comparing the magnitude of the BOLD responses to emotional and neutral movie segments in these regions. Significantly stronger responses to emotional segments were revealed in most (10/12) of the global motion ROIs (FDR corrected for multiple comparisons). Similarly, both local motion ROIs showed significantly stronger responses to emotional than to emotionally neutral content.

**Table 2:** Areas showing significant BOLD responses to global and local motion.

Region	Side	BA	X	Y	Z	t	p
***Global Flow*** ** > ** ***Rest***							
Middle Occipital Gyrus	L	18	−24	−82	−2	26.62	<0.00001
Superior Temporal Gyrus	R	38	36	20	−23	7.86	<0.00001
Inferior Frontal Gyrus	R	45	54	23	13	5.01	0.000395
Middle Frontal Gyrus	R	6	30	−10	46	10.53	<0.00001
Thalamus	R		9	−28	1	7.83	<0.00001
Cingulate Gyrus	R	31	15	−28	40	4.36	0.001128
Superior Frontal Gyrus	R	10	9	68	7	4.42	0.001024
Cerebellum (Culmen)	R		0	−46	4	5.26	0.000267
Thalamus	L		−18	−28	7	5.51	0.000184
Posterior Cingulate Gyrus	L	31	−12	−28	43	4.79	0.000559
	L	31	−21	−61	16	4.20	0.001489
Middle Frontal Gyrus	L	6	−21	−10	58	5.56	0.000169
***Local Flow*** ** > ** ***Rest***							
Left Cuneus	R	17	9	−97	7	11.13	<0.00001
Right Cuneus	R	17	−12	−97	4	9.80	<0.00001

Corrected for multiple comparisons both at the voxel level (FDR < 0.05) and at the cluster level (at p < 0.05). BA, Brodmann area.

**Figure 4:**
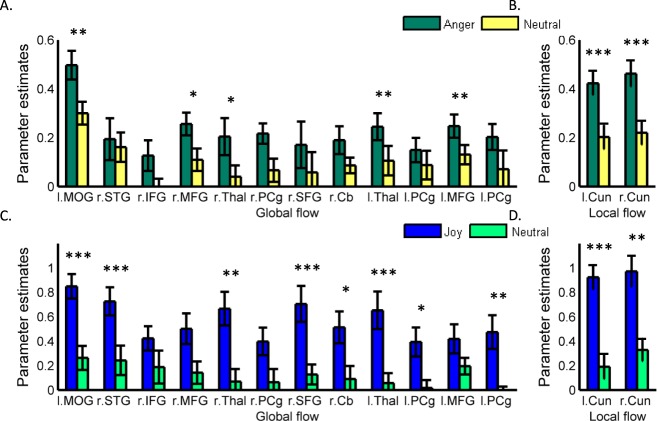
Brain activation related to processing of discrete emotions in Region of Interest tuned to global and local flow. ROI analysis comparing brain activity elicited by anger and joy, relative to neutral content, was performed for all regions responsive to global (**A**,**C**) and local (**B**,**D**) flow. * indicates statistical significance at P < 0.05; **P < 0.01; ***P < 0.001. All abbreviations are as in previous figures.

So far, our analyses focused on links between motion and emotion perception, where ‘emotion’ was defined irrespective of the actual emotion portrayed in the observed movie segments. The major reason behind using this inclusive definition was twofold. First, our objective was to find general principles that link motion and emotion perception. Second, from a methodological point of view, our sample of clips depicted complex emotional contents, which- similarly to emotions, as perceived in real life, mostly included more than one discrete emotion (see Materials and Methods). Still, we wanted to assess whether the links between local and global motion and emotion perception are retained when discrete emotions are analyzed (i.e. when each clip portrayed one single emotion). Indeed, repeating the ROI analysis, as reported above, of regions tuned to global and local flow (Fig. 4), revealed stronger responses to anger (Fig. 4A) and joy (Fig. 4C), relative to neutral content, in most of the global and local motion ROIs. These results further stress the significance of global versus local motion signals in the differentiation of emotion and neutral content, also when analyzing the perception of discrete emotions such as joy and anger.

### Contribution of low-level scene features

Since global motion was generally more pronounced than local motion in both emotional and neutral clips (Fig. 1), one possibility that we had to address is that low-level visual features in scenes with more global motion may have contributed to the stronger modulating role, reported thus far for global motion. We thus next determined if emotion perception under naturalistic viewing conditions solely relies on lower-level scene statistics, as the results so far may imply. To that effect, we carried out a control experiment where all the movie stimuli- emotional and neutral- were inverted and displayed to a subset of the subjects (n = 8) in an upside-down orientation. Contrasting inverted emotional clips against inverted neutral clips (using the same significance threshold as above) yielded no significant clusters of activation. We can thus rule out the possible interpretation that low-level scene statistics was the sole source of information driving brain activity during movie viewing. Given that the emotional movie clips contained more global motion than neutral clips (Fig. 1), these results also suggest that the amount of global motion per se did not merely induce stronger or more widespread brain responses. Were this the case, displaying the inverted movies should have resulted in a similar difference between emotional and neutral clips.

### Contribution of movie shot type

In attempting to establish the contribution of global and local motion to emotion perception we tested the possible influence of an alternative modulating cinematographic cue, namely the type of shot depicted in emotional and neutral clips. Manipulation of shot types is a known and strong cinematographic technique used for manipulating emotional responses in movie viewers^1^. Different shot types were shown to constitute a major feature in inducing affective response in movie viewers^11^. We thus tested whether, in the sample of clips analyzed here, emotional and neutral clips differed in the type of shots that were included in each clip. Our analysis, in particular, focused on the amount of static and dynamic shots within each movie segment (Supplementary Table 1), testing if it could predict whether the clip was classified by our observers as being emotional or neutral. A logistic regression analysis revealed that neither static (B = 0.536, ns), nor dynamic shots (B = −0.454, ns) could predict whether movie segments were emotional or neutral.

## Discussion

Taken together, the results suggest that motion information plays a substantial role in modulating the neural responses to emotional content under the rich and unconstrained visual processing settings associated with movie viewing. The results reveal that brain activity in areas that showed preferential responses to emotional compared to neutral content was strongly linked over time with frame-wide variations in global motion fields, induced by large camera movements, and to a lesser extent with local motion information mainly related to object and human movements. Similarly, stronger responses to emotional content were seen within ROIs whose activity was strongly attuned to global and local motion over time. Neither global nor local motion by themselves were the sole drivers of emotion processing. Thus, motion signals, particularly global motion, appear to interact with or modulate the perceptual responses to other movie features, such as narrative and content, and to emotional cues such as facial expressions, or affective gestures.

The results indicate that a network composed of frontal, occipito-temporal, parietal and cerebellar regions was more strongly responsive to emotional, relative to emotionally neutral movie segments. The naturalistic and unconstrained stimuli used in the current study contained dynamic full-body depictions of emotion, which were rich in detail and context. In this respect, the results are in strong agreement with previous results concerning the observation of dynamic and static^12,13^ bodily depictions of emotion and join these results, and others^4,5,14,15^ in delineating the rich sources of information used by the brain during emotion perception. Our results, in particular, confirm that dynamic, full-body portrayals of emotion, as contained in the movie stimuli, recruit emotion processing areas. These include the fusiform gyrus^12,13^ along with structures in the dorsal stream implicated in visual processing which may contain emotion representations^16,17^ and structures such as the STG, supramarginal gyrus and IFG, known to be crucial for the perception of action and biological motion^18–20^. The results also documented stronger activation in posterior cerebellum in response to emotion, consistent with earlier reports on its contribution to the processing of primary emotions^21^. Notably, the distributed pattern of activation observed here in response to emotional content is in agreement with emphasizing the network perspective of emotion representation^4^ where emotion perception is viewed as a product of sensory, emotional and motor system interactions.

Our results reveal a consistent modulating role for local and global flow fields in emotion perception. Converging evidence from animal neurophysiology and human imaging studies implicated regions along the medial temporal and dorsomedial temporal lobes in the encoding of visual information associated with optic flow^22^ and more specifically self-motion^6–8^. Global flow fields are used by the visual system for estimation of self-motion^23^. It has been suggested that while area MT specializes in a more general analysis of motion signals, area V6 appears to be more specifically involved in distinguishing between object and self-motion^7,8^. However, in agreement with earlier reports^3^ our results revealed spatially segregated brain responses to global and local motion, with the former eliciting a more distributed response, not confined to areas MT or V6, but rather encompassing regions in occipito-temporal, parietal and frontal cortices. It is important to note that our results do not suggest that global (or local) motion induced a selective emotional response. Rather, our results suggest that activity in emotion responsive brain regions was more strongly associated with the amount of global motion in the scene, rather than with that of local motion. Our use of unconstrained movie clips prevented us from assessing whether global or local motion are capable of inducing emotion selectivity in the brain since movie frames continuously depicted both local and global motions (Fig. 1B). Experimental manipulations of these types of motion would be needed in order to test if these different motion types can induce a selective emotional response.

It was recently shown that by reducing the distance between observer and observed agent, camera movements, and by inference, global motion cues, preferentially engage the motor system during observation of goal directed action^9^. Our findings add to these observations, suggesting that global motion cues are also a key modulator of brain responses to observed emotion. As global flow fields are experienced during self-motion^6–8^, we suggest that these modulatory effects may operate through the induction of an illusory self-motion percept in subjects. Recent years have seen significant rise in interest in the influence that behavioral states have on sensory processing^24,25^, including the effects of movement^26,27^. For instance, in the mouse, transition from a still position to running strongly affects visual responses in primary visual cortex, without changes in spontaneous firing or stimulus selectivity^27^. Technological constraints rendered similar investigation of movement-dependent sensory processing in humans problematic. By using naturalistic stimuli and quantifying the degree of global motion we were able to overcome these limitations, revealing consistent and dynamic modulation of visual responses to affective stimuli induced by camera movement, which we suggest may reflect the influence of self-movement cues on emotion perception.

Links between emotion and action have been a central theme in theories of emotion^28,29^. In an influential account, Darwin postulated an adaptive capacity for perceived emotion in motivating observers to act and respond^30^. As camera movements move the viewer towards and away from the scene they may generate an approach and avoidance visual context^31,32^, which is fundamental to emotion processing^33^. In addition, assuming that camera movement indeed induces illusory self-motion, this may create more immersive viewing settings^34^, leading to more intense emotional responses in viewers^35^. As attention has been shown to modulate global motion perception^36^, our results may also reflect enhanced attentional capture by global motion signals. Thus, by generating a more immersive viewing environment and through interactions with attentional mechanisms, global motion may keep viewers more engaged in the movie. Future research could confirm these possibilities, by using immersion rating measures, and assessing attentional capture in viewers in conjunction with analysis of the contribution of global versus local motion to emotion perception.

Circuits implicated in the embodied simulation of others’ emotional states contribute to the generation of emphatic responses during movie viewing, mediated by interactions with limbic networks^37^. Thus, through such large-scale network interactions emotional movie segments may further contribute to emotion perception. It was suggested that camera movements may simulate the virtual presence of viewers within the observed movies^38^. By simulating self-motion, camera movements may form immersive viewing states that interact with other sources of visual information and with the processing of the underlying narrative of the scene, leading to the generation of emotional responses in viewers. As such, the manipulation of camera movements may join other cinematographic techniques which have evolved over the years to render movies to be more compatible with the natural dynamics of human vision and attention^39^. Additional work is needed in order to confirm these suggestions.

Taking advantage of the rich sources of motion information depicted in movies, our study revealed how motion contributes to emotion processing under viewing conditions that approximate those experienced in real life. The naturalistic paradigm utilized here enabled us to identify a strong role for global motion signals, closely resembling those experienced during self-motion. Thus, using movie stimuli provided a unique opportunity to assess the contribution of self-motion to emotion perception. However, the unconstrained and uncontrolled visual paradigm we have used also warrants caution in the interpretation of the results. For instance, our analysis of local motion did not specifically isolate the contribution of human motion to the perception of emotion, as the local motion signals captured non-human motion as well. Our approach also did not allow us to assess interactions between local and global motion. Our study also cannot rule out the contribution of learned responses in our subjects and their acquired experience with respect to cinematic conventions. In addition, our analysis did not currently focus on the specific kinematic features of global and local motions, which may strongly attenuate the perceptual response to visual motion^40–42^. Future studies may use more controlled paradigms, or alternatively other analytical approaches, while using naturalistic stimuli to further delineate the contribution of motion signals to emotion perception.

To summarize, our results suggest that motion signals, particularly global motion, play a substantial role in modulating the neural responses to emotional content under the rich and unconstrained visual processing settings of movie viewing. These results confirm the validity of practices which have been used for many years by movie directors and cinematographers, while joining earlier reports in demonstrating the complex and multifaceted nature of emotion perception.

## Materials and Methods

### Participants

Twelve healthy volunteers participated in the study (mean age = 28.9 ± 4.9; 6 females). All participants were right handed and had normal or corrected to normal vision. Written informed consent was obtained from all volunteers and the Institutional Review Board of the Tel-Aviv Sourasky Medical Center approved all the procedures used. All procedures were performed in accordance with the relevant guidelines and regulations.

### Stimuli

Fourteen 1-minute clips, portraying either emotional or emotionally neutral content were used in the study. The clips were extracted from the movies: ‘The Godfather I’, ‘The Shining’, ‘Dog Day Afternoon’, ‘Scarface’ and ‘One Flew Over the Cuckoo’s Nest’ (for a full list of clips see Supplementary Table 2). The 1- minute segments were extracted in full, with no additional editing manipulations. In order to focus our work on the contribution of motion signals to emotion perception, the clips were extracted (and eventually played inside the scanner) with no sound. The clips were chosen from a larger pool of clips based on the assessment of six observers (mean age = 29.5, 4 females, none of which participated in the main fMRI experiment). Observers were asked to carefully observe all movie clips, and to freely indicate, for each clip, whether it contained an emotional expression, identify specific emotional expressions whenever those occurred and indicate the degrees of intensity, and authenticity of each identified emotional expression. The clips chosen to serve as stimuli in the subsequent fMRI experiment were the ones that received the highest intensity and authenticity ratings from our subjects. Overall, the clips depicted the following emotions: anger (3 clips), joy (1 clip) or a combination of anger and another emotion, either joy or fear (3 clips). Emotionally neutral clips were all extracted from the same movies and depicted human movements and actions, but no emotional content.

### Functional MRI experiment

Emotional and neutral clips were displayed to subjects inside a 3T Siemens scanner. The stimuli were presented using a projector (Epson PowerLite 74C, resolution = 1024 × 768), and were back-projected onto a screen mounted above subjects’ heads, and seen by the subjects via an angled mirror. The stimuli were delivered using the software Presentation (www.neurobs.com). The distance from the projector to the mirror was 96 Cm and approximately 14 Cm from the mirror to subjects’ eyes. The size of the film frame inside the scanner (in degrees) was approximately 1.6° (width) 1.2° (height).

The clips were displayed, interleaved by 12-sec periods of fixation in one continuous run containing 14 clips (7 emotional and 7 neutral clips). The short fixation periods allowed the fMR signal to reach stable baseline levels before each clip was shown. Similarly to previous experiments which utilized a naturalistic viewing paradigm^10,43,44^, subjects were not given any task, and were simply asked to freely observe the clips. In addition to the main experiment, we also ran a control experiment (n = 8; all participated in the main experiment as well) where the same clips were inverted and displayed upside-down.

### Imaging setup

Blood Oxygenation Level Dependent (BOLD) contrast was obtained with a gradient-echo echo-planar imaging (EPI) sequence (repetition time = 2,000 ms; echo time = 30 ms; flip angle = 75°; field of view, 256 × 256 mm; matrix size, 90 × 90). The scanned volume included 32 oblique-axial slices of 3 mm thickness. In addition, T1-weighted whole-brain magnetization-prepared rapid-acquisition gradient echo (MPRAGE) anatomical scans (176 slices of 1 mm thickness) were acquired from all subjects.

### Data analysis

Preprocessing of imaging volumes was performed with Brain-voyager QX (Brain Innovation). The first 6 volumes were discarded in order to control for T1 equilibration effects. The remaining volumes were corrected for head-motion artifacts, and the data were high-pass-filtered to remove low-frequency drifts (up to five cycles per experiment) and spatially smoothed using a 6 mm full-width-half-maximum Gaussian kernel. The functional images were then superimposed on the anatomical images and incorporated into 3D data sets through trilinear interpolation. The complete data set was transformed into Talairach space. A general linear model (GLM)^45^ was then fitted to the functional data, whereby each 1-minute movie segment (emotional or neutral) served as a predictor in the model. All regressors were modeled as box-car functions convolved with the hemodynamic response function. A hemodynamic lag of 3 to 6 seconds was assumed and verified for each subject. Group results are based on a GLM, where subjects serve as a random effect and movie type (emotional or neutral) as a fixed effect. Correction for multiple comparisons was applied both at the voxel level (with a False Discovery Rate^46^, or FDR, set at 0.05) and at the cluster level (at p < 0.05) using the cluster threshold estimation plugin for BrainVoyager.

### Scene analysis

Methods used for scene analysis followed those described in Bartels *et al*.^3^ and are described in full there. All analysis was performed with MATLAB. The main aim of this part of the analysis was to quantitatively track scene-wide changes in motion information and partition these changes into components related to local and global motion. The analysis comprises two major steps:

1. Computation of optic flow for motion detection
2. Partitioning of motion information into global and local components

The first step we applied to analyze the scenes observed by subjects was computation of optic flow. We extracted dense optic flow fields using an accurate and robust estimation technique^47,48^. The result of the first phase provides information on pixels that changed value due to motion in the scene, on a frame by frame basis, spanning the entire movie display (and due to, for example, light changes). However, a pixel in the scene can move either as a result of camera movement (global motion) which affects most of the pixels in the scene or as a result of object or human movements (local motion). Therefore, in the second step of scene analysis motion was attributed to global or local components. Following Bartels *et al*.^3^ we first generated a pool of “ideal” flow fields based on each of the real flow fields, r, spanning the whole display, g. For these purposes ideal flow fields were defined as fields with a single uniform motion trend (e.g., upwards) spanning the entire frame. We then projected the extracted flow on the ideal flow database to find the underlining flow responsible to the majority of the movement in the frame^3^. Then, once the global flow of the frame was known, it was possible to extract local flow by subtracting global from total flow. All measurements were averaged across the whole movie display.

### Shot and camera movement analysis

To assess whether the type of camera movement included in the various clips contributed to the classification of the clips as emotional or neutral we had a professional cinematographer classify the types of shots depicted in each movie. Each shot was thus classified as being either static or dynamic, and the type of movement (pan, tilt etc) was additionally determined in the dynamic shots. The cinematographer was blind to the classification provided by our observers of the movie clips as being either emotional or neutral.

### Links between f low and BOLD time series

Links between emotion-related brain activation and the two motion signals (global and local) were calculated based on a two-step procedure. ROIs were first placed at the peak location of each of the regions that showed preferential responses to emotional relative to neutral clips. BOLD time series were then extracted from each of the ROIs, for each of the subjects. On the first step of the procedure, we regressed the BOLD signals taken from each ROI with the local and global flow signals, which were convolved with a canonical hemodynamic response function (HRF), filtered using a 2nd order Butterworth filter and down-sampled in time. Due to strong auto-correlations in the constituting time series, this analysis was based on a generalized least square (GLS) regression with an autoregressive correlation structure (first order AR1 autocorrelations were modeled)^49^. The analysis was performed in ‘R’ (https://www.r-project.org/), using the ‘nlme’ package. On the second step of the procedure, regression coefficients derived from the single-region models (as described above) were compared using paired two-tailed t tests, corrected for multiple comparisons using FDR correction (FDR thresholds were computed using the ‘fdr’ function in MATLAB). We also tested whether the coefficients were significantly different than ‘0’ using two-tailed one sample t tests, FDR corrected for multiple comparisons. All signals were z-transformed for visualization purposes. The two step procedure was applied in the analysis of emotional vs. neutral clips, and not in the analysis of the more discrete emotions, since very few clips portrayed discrete emotions Supplementary Table 2, resulting in time series which were too short for this type of analysis.

### General linear model analysis with flow signals as regressors

In this analysis the filtered and down-sampled local and global flow signals were convolved with an HRF and served as regressions in a separate GLM analysis. Group statistical maps were then generated for each type of flow signal and were corrected for multiple comparisons, both at the voxel level (with an FDR set at 0.05) and at the cluster level- (at p < 0.05) using the cluster threshold estimation plugin for BrainVoyager. ROIs were then defined at the peak of each cluster in these maps. The ROIs were subsequently used to compare brain activation elicited by emotional as compared to neutral clips; and by the two discrete emotions (anger and joy), also when compared to neural clips. The comparisons were based on paired two-tailed t tests, with the threshold of significance adjusted using DFR correction.

### Data availability statement

The datasets are available from the corresponding authors on reasonable request.

## Electronic supplementary material

Supplementary Materials
