# Supplementary material for: Motion cues modulate responses to emotion in movies
Source: Sci Rep. 2018 Jul 18;8:10881. doi: 10.1038/s41598-018-29111-4 (PMC6052113; doi:10.1038/s41598-018-29111-4)
Supplement: Supplementary file 1 — Supplementary Materials [file 41598_2018_29111_MOESM1_ESM.docx]

Supplementary Materials for:

**Motion cues modulate responses to emotion in movies**

Eran Dayan, Avi Barliya, Beatrice de Gelder, Talma Hendler, Rafael Malach & Tamar Flash

**Supplementary Table 1**

| **Clip** | **static** | **movement** | **Total** |
| --- | --- | --- | --- |
| Emotional 1 | 12 | 8 | 20 |
| Emotional 2 | 5 | 7 | 12 |
| Emotional 3 | 1 | 5 | 6 |
| Emotional 4 | 10 | 11 | 21 |
| Emotional 5 | 7 | 4 | 11 |
| Emotional 6 | 2 | 10 | 12 |
| Emotional 7 | 1 | 2 | 3 |
| Neutral 1 | 4 | 2 | 6 |
| Neutral 2 | 3 | 7 | 10 |
| Neutral 3 | 5 | 6 | 11 |
| Neutral 4 | 14 | 1 | 15 |
| Neutral 5 | 6 | 2 | 8 |
| Neutral 6 | 1 | 3 | 4 |
| Neutral 7 | 3 | 3 | 6 |

Static and dynamic camera movement, in each of the 14 analyzed clips.

**Supplementary Table 2**

| **Onset in Movie** | **Emotion/Neutral** | **Emotions Portrayed** |
| --- | --- | --- |
| *One Flew Over the Cuckoo's Nest (1975)* | | |
| 13:45 | Neutral |  |
| 30:03 | Neutral |  |
| 44:22 | Emotional | Joy, anger |
| 1:05:34 | Emotional | Joy |
| *The Shining (1980)* | | |
| 2:57 | Neutral |  |
| 1:02:09 | Emotional | Anger |
| *Scarface (1983)* | | |
| 2:01:56 | Neutral |  |
| 2:17:25 | Emotional | Anger |
| *Dog Day Afternoon (1975)* | | |
| 5:34 | Neutral |  |
| 29:45 | Emotional | Anger |
| 45:50 | Neutral |  |
| 1:29:07 | Emotional | Sadness, anger |
| *The Godfather (1972)* | | |
| 22:16 | Emotional | Anger, joy |
| 34:11 | Neutral |  |

Movie segments used in the current study. The clips were presented to subjects (order was randomized), interleaved by 12-sec periods of fixation in one continuous run
